# The Multiple Roles of B Lymphocytes in the Onset and Treatment of Type 1 Diabetes: Interactions between B Lymphocytes and T Cells

**DOI:** 10.1155/2021/6581213

**Published:** 2021-11-03

**Authors:** Yangfan Xiao, Chao Deng, Zhiguang Zhou

**Affiliations:** ^1^Clinical Nursing Teaching and Research Section, Department of Anesthesiology, and Anesthesia Medical Research Center, The Second Xiangya Hospital of Central South University, Changsha 410011, China; ^2^National Clinical Research Center for Metabolic Diseases, Department of Metabolism and Endocrinology, and Key Laboratory of Diabetes Immunology, Ministry of Education, The Second Xiangya Hospital of Central South University, Changsha 410011, China

## Abstract

Although type 1 diabetes is thought to be an organ-specific autoimmune disease, mediated by effective CD4^+^ and CD8^+^ T cells, it has recently become clear that B cells participate in the initiation and progress of this disease. Indeed, B cell deletion can prevent or reverse autoimmune diabetes in nonobese diabetic mice and even result in partially remaining *β* cell function in patients with new-onset type 1 diabetes. This review summarizes the dual role of B cells in this process not only of pathogenic effect but also of immunoregulatory function in type 1 diabetes. We focus on the impact that B cells have on regulating the activation, proliferation, and cytokine production of self-reactive T cells along with regulatory T cells, with the aim of providing a better understanding of the interactions between T and B cells in immunopathogenesis and improving the efficacy of interventions for clinical practice.

## 1. Introduction

Type 1 diabetes (T1D) is regarded as an organ-specific autoimmune disease, during which innate and adaptive immune cells comprising CD4^+^ and CD8^+^ T cells, B cells, and antigen-presenting cells (APCs) are involved in a dynamic progression of inflammation of the islet contributing to the loss of pancreatic *β* cells [1]. Accumulating evidences show that B cells have participated in the initiation and progress of T1D [2], with immunological heterogeneity that differed significantly by age at diagnosis [3, 4]. CD4^+^ T cells clearly offer help to B cells and promote humoral immune responses. However, the inverse condition that B cells regulate T cell-mediated autoimmune destruction is not widely recognized in T1D.

**Figure 1:**
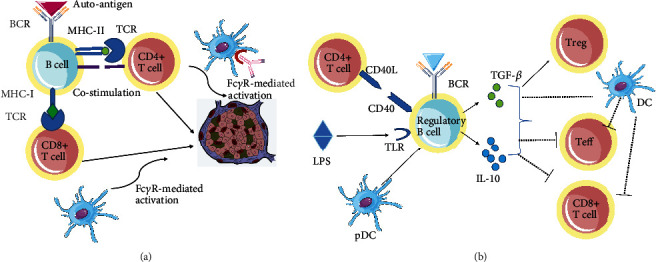
Mechanism of B cell involvement in self-reactive T cell-mediated *β* cell destruction. (a) The imbalance of pathogenic and regulatory B cells contributes to a loss of immune homeostasis. Autoreactive B cells may specifically recognize autoantigen via unique BCR and present to autoreactive T cells through MHC-peptide-TCR, with increased expression of MHC classes I and II as well as elevated B7-1 and B7-2 levels. Autoantibodies produced by activated B cells enhance islet-reactive T cell activation through an Fc*γ*R-mediated manner. (b) Regulatory B cells can be induced by various stimuli including LPS, anti-IgM F(ab′)_2_ antibody, CD40L, and plasmacytoid dendritic cells (pDC). By contrast, the activated regulatory B cells produce IL-10, which suppresses the inflammatory potential of effector T cells, alters the activity of antigen-presenting dendritic cells, and promotes regulatory T cell development and expansion. BCR: B cell receptor; LPS: lipopolysaccharide; MHC: major histocompatibility complex; TCR: T cell receptor; TLR: toll-like receptor.

B lymphocytes have a crucial role in the etiology of T1D as antigen-specific presenting cells [5–7], expressing high levels of MHC classes I and II and elevated costimulatory molecules [7, 8] and autoantibody secretors [9, 10], secreting regulatory cytokines [11–13]. Indeed, B cell depletion in nonobese diabetic (NOD) mice, either through genetic target [14], monoclonal antibody treatment [15–17], or blockade of B cell–activating factor (BLyS/BAFF) by anti-mouse BLyS mAb or B cell maturation antigen (BCMA)-Fc [18, 19], could prevent and even reverse autoimmune diabetes. In line with these findings, a phase II randomized controlled trial, deletion of B lymphocytes with rituximab, can delay the loss of *β* cell function in patients with new-onset T1D [20]. These studies indicated that B cell depletion inversely has an effect on CD4^+^ and CD8^+^ T cell response and regulatory T cells (Tregs). Besides, naive or activated B cells also exhibit regulatory function to maintain tolerance to islet autoantigens or prevent T1D in NOD mice [11–13]. Understanding these cellular interactions between B and T cells will offer vital insight into the immune pathogenesis and improve the efficacy of interventions for clinical practice. Here, we summarize the dual role of B cells in this process, not only of effective function but also of immunoregulatory effects in T1D. We focused on the impacts that B cells have on regulating the activation, proliferation, and cytokine production of self-reactive CD4^+^ T cells as well as the mechanisms aforementioned, and we also discussed the effect of B cell deletion on CD8^+^ T cells and Tregs (Figure 1).

## 2. Defective B Cell Tolerance in T1D

Emerging evidence showed that the disruption of B cell tolerance mechanism is a major contributor to T1D [21, 22]. Although islet antigen-reactive B cells are silenced by central and peripheral tolerance mechanisms in healthy subjects, they may become activated and result in the development of T1D [21, 23]. There is an elevated B cell response to autoantigens in the peripheral blood of subjects with new-onset, but not long-standing, T1D [23]. Smith et al. [21] also found loss of anergic B cells in patients with prediabetes and newly diagnosed T1D compared with healthy controls. They further reported that high-risk HLA alleles and a subpopulation of non-HLA risk genotypes, related to B and T cell development and function are correlated with loss of anergy [24]. These results indicated that loss of B cell anergy precedes T1D onset and maybe gives rise to the development of autoantibodies and disease progress. Insulin-reactive 125Tg B cells favor T cell-mediated T1D in NOD mice, in spite of being anergic to B cell mitogens as well as T cell-dependent immunization. These B cells stimulate both experienced and naive CD4^+^ T cells more efficiently compared with naive B cells, suggesting the pathological correlation of anergic B cells in T1D [22]. Intriguingly, selectively targeting insulin-specific B cell receptors by mAb123 depletes insulin-binding B cells and protects NOD mice from T1D [25].

### 2.1. B Cells as Producers of Islet Autoantibodies

Although the presence of circulating islet autoantibodies in patients with T1D raises the possibility of a pathogenic role, the significance of autoantibodies remains controversial. Compared to B cell-deficient NOD mice, transgenic mIg.NOD mice in which B cells express membrane-bound but not secreted IgM develop diabetes, suggesting that the production of islet autoantibodies is not the essential contribution of B cells to diabetes development [26]. However, Diabetes Prevention Trial–Type 1 found that autoantibody number can help identify human T1D risk [27]. Harbers et al. [28] showed that adoptive transfer of antibodies against islet-expressed ovalbumin promotes activation of islet-responsive CD8^+^ T cells and induces autoimmune diabetes in NOD mice, which depends on the presence of activating Fc receptors for IgG and crosspriming dendritic cells (DCs) [28, 29]. Besides, one recent study found that anti-islet autoantibodies enhance the expansion of islet-reactive CD4^+^ T cells through an FcgR-mediated manner and promote progression to autoimmune diabetes, which is in agreement with previous findings [9, 28]. The influence of maternally transmitted autoantibodies on the development of autoimmune diabetes is still ambiguous. Maternal transmission of islet autoantibodies promotes autoimmune diabetes in NOD mice [30]. Furthermore, elimination of maternally transmitted autoantibodies protects NOD mice from autoimmune diabetes [30]. In contrast, human research demonstrated that fetal exposure to islet autoantibodies has significantly lower risks for developing multiple islet autoantibodies and diabetes [31]. It seems that in NOD mice, B cells promote *β* cell destruction and disease progression primarily through presentation of antigen to autoreactive T cells.

### 2.2. B Cells Present Autoantigen and Provide Costimulation Signal to T cells

B lymphocytes invade the mouse and human pancreas in the early stages of inflammation of the pancreatic islets [2, 3]. These B cells are antigen-experienced and competent to induce islet-infiltrating T cell proliferation and become a permanent component of the pancreatic infiltration once formed [32]. B lymphocytes play a significant role in the development of T1D, including autoantigen-specific presentation [5–7, 14] and costimulation with CD4^+^ T cells [7, 8]. *μ*MT^−/−^ mice have no mature B cells in the periphery due to the introduction of a functionally inactivated immunoglobulin *μ* heavy chain gene, deleting IgM and resulting in B cell developmental arrest in the bone marrow [33]. B cell-deficient NOD.*μ*MT^−/−^ mice have a normal number of T cells but are protected from apparent hyperglycemia, suggesting an essential effect of B cells on the initial activation of diabetogenic T cells in NOD mice [14]. T cells from B cell-insufficient NOD.*μ*MT^−/−^ mice had no proliferative response to GAD65 compared with those from B cell-adequate NOD mice [6]. Similarly, T cells from NOD mice demonstrated more potent proliferation to B lymphocytes rather than other APCs in vitro, implying B cells are preferential APCs to autoreactive T cells [34], especially when the antigen is limited [35]. Besides, T cells from diabetes-protective NOD.*μ*MT^−/−^ mice could not respond to GAD65, no matter presented by B cells or other APCs [34]. Another study showed an essential necessity for B and T cell cognate interplay for the activation of self-responsive CD4^+^ T cells [5]. When B cell-defective NOD.*μ*MT^−/−^ mice were reintroduced with splenic B cells, which carried MHC class II haplotype, IA^g7^, diabetes was prevalent again [5]. However, when NOD.*μ*MT^−/−^ mice were reorganized with IA^g7^-defective B cells, diabetes was prevented [5]. Therefore, the singular B cell-specific depletion of MHC class II IA^g7^ prevented diabetes onset, in spite of the presence of IA^g7^ on functionally capable non-B cell APCs.

Although CD4^+^ and CD8^+^ T cells invade islets, *β* cell destruction is considered mainly due to CD8^+^ cytotoxic T cells [36]. There is also the probability that B lymphocytes might be significant for self-responsive CD8^+^ T cell activation [37]. Diabetes onset is subverted in mixed bone marrow chimeras, in which B cell-specific MHC class I is defective [7]. This diabetogenic function rely on MHC class I expression of B cells, implying that it proceeds through B lymphocyte crosspresentation of islet autoantigen to self-responsive CD8^+^ T cells [7]. TNF*α*-*μ*MT^−/−^ mice delayed significantly the progression of diabetes compared to wild-type TNF*α*-*μ*MT^+/+^ mice. Of interest, diabetes progress in TNF*α*-transgenic NOD mice is CD8^+^, but not CD4^+^, T cell dependent. B cell depletion results in a general reduction of CD8^+^ T cells and suppresses their differentiation into cytotoxic T-lymphocytes in inflamed islets [38]. In parallel with this study, Marino et al. showed that B cell maturation antigen- (BCMA-) Fc treatment blocks the support for CD8^+^ T cell differentiation and survival [19]. Islet-infiltrated B lymphocytes have increased expression of MHC I and II and elevated B7-1 and B7-2 levels, in accordance with increased T cell activation [7, 8]. Therefore, B cells might have a crucial role in T1D through activating self-reactive CD4^+^ T cells via direct cognate interactions and favoring CD8^+^ T cell survival and differentiation.

## 3. Altered B Cell Compartment in T1D

Indeed, altered peripheral B cell subsets have also been observed in subjects with T1D. These changes comprised a decrease in C-X-C motif chemokine receptor 3, transitional B cells, receptor for B cell activating factor, and TACI expression [47–49]. We also reported a decreased frequency of regulatory CD19^+^CD5^+^CD1d^hi^ cells in T1D than latent autoimmune diabetes in adults and type 2 diabetes [50]. Recent researches have expanded these outcomes through detecting B lymphocytes infiltrated in the islet and pancreatic lymph nodes at different stages of T1D. The researchers revealed two kinds of distinct patterns of insulitic lesions, which were defined as CD20^hi^ (more B cell infiltration) and CD20^lo^ (less B cell presence) [4]. It is relevant to mention that, in humans, B cells rank second right after CD8^+^ T cells, for abundance in the islet infiltrates of T1D patients in “CD20^hi^ hyperimmune patterns,” showing more extensive beta-cell destruction [4]. Patients diagnosed with T1D before the age of 7 years always exhibit a CD20^hi^ islet profile, while subjects diagnosed beyond 13 years of age are consistently identified as CD20^lo^ phenotype. Individuals diagnosed between the age of 7 and 12 years could belong to either group. These results revealed that the two kinds of patterns of disease are distinguishingly invasive. In support of the hypothesis, individuals with the CD20^hi^ islet profile display a more rapid decline of *β* cell function than those with the CD20lo phenotype [4]. Of interest, another histological analysis of rare pancreatic lymph nodes found that patients with new-onset T1D have fewer numbers of primary B cell follicle frequencies and follicular dendritic cell networks than those with long-standing T1D and healthy controls [51].

### 3.1. B Cell Depletion in T1D

Of interest, depletion of B cells by monoclonal antibodies targeting cell surface CD20 [15, 16] or CD22 [17] and inhibiting the pivotal B cell-activating factor (BAFF) through BCMA-Fc [19] or anti-BAFF mAb [18] has similarly protective effect in the NOD mice. These studies provide forceful evidence that B cells indeed have an effect on pathogenic and regulatory T cell responses (Table 1). Rituximab treatment before disease onset had higher numbers of Foxp3^+^ T cells and reduced interferon- (IFN-) *γ* and interleukin- (IL-) 17 by T cells [15]. Transfer of CD4^+^ T cells from these mice or cotransfer repopulated B cells with autoreactive T cells suppressed diabetes development [15], implying that depletion of B cells generated both B and T cells with regulatory functions. An induction of Foxp3^+^ T cells was also discovered in NOD mice following treatment with CD22-specific monoclonal antibodies [17] or BCMA-Fc [19]. B cell-targeted therapies also inhibit self-responsive CD4^+^ [15–19] and CD8^+^ [16, 18] T cell reactions. Interestingly, before B cell reconfiguration, autoantigen-specific CD4^+^ T cells respond more firmly to islet-specific antigens ex vivo [39, 40], which identifies clinical responders to rituximab treatment in patients with T1D [40]. In addition, the newly emerging B cells after depletion prevented diabetes development when transferred with pathogenic T cells into NOD-SCID mice [15, 17]. This enhanced regulatory function seems to depend on direct cell contact other than IL-10 production [39].

Collectively, the evidence aforementioned indicates that there is a special interplay between B lymphocytes and T cells in the development of diabetes. This bidirectional B and T interactions have been abrogated by depletion of B cells **(**Table 1**)**. These data have contributed to a vital interest in the use of B cell depletion applied in a randomized, double-blind, controlled, phase II clinical trial in recent-onset subjects with T1D. A four-dose course of rituximab showed higher C-peptide area under the curve for up to a year and reduced daily insulin requirement [20]. Rituximab obviously inhibited insulin autoantibody but had less effect on glutamic acid decarboxylase antibody, insulinoma-associated protein-2 antibody, and zinc transporter 8 antibody. After two-year follow-up, the declined rate of C-peptide was comparable but shifted by 8.2 months between groups [41]. It does not seem to overcome defective B cell tolerance checkpoints fundamentally, as the frequencies of self-reactive B cells remained increased in the peripheral blood one year after rituximab treatment [42]. It is crucial to notice that B cell entry into islets downregulates CD20 expression [43]. In humans, CD20^hi^ and CD20^lo^ B lymphocytes infiltrate the inflamed islets, which represent differentially aggressive islet inflammation associated with the age of onset [3, 4]. Taken together, these findings may complicate the efficacy of B cell-based therapy.

In addition, B cell receptor specificity for islet autoantigen is pivotal for distinct B cell function in the disease progress [22, 44]. Selective targeting anti-insulin B cell receptors exhaust autoantigen-specific B cells in vivo and prevent T1D [25, 45]. Recently, a certain subpopulation of B cells, namely, B1a cells, plays a critical role in the initiation of diabetogenic T cell response through activation of pDCs [2], as depletion of peritoneal B1a cells delays diabetes onset [46].

## 4. Regulatory B Cells Prevent Disease Pathogenesis

It has become clear that the development of autoimmune diabetes is attributed to an imbalance of effective and regulatory immune cells, which contributes to a disruption of immune homeostasis. A defect in immunoregulatory pathways may be a major determinant in the initiation and progression of T1D. In support of this concept, two separate phase I studies showed that transfusion of autologous Tregs delays remission in patients with newly diagnosed T1D [52, 53].

Accordingly, B lymphocytes have been reported to play an immunoregulatory role in T1D (Figure 1). Various regulatory B cells (Bregs) have been shown to proceed through anti-inflammatory cytokines IL-10 or transforming growth factor- (TGF-) *β*. Lipopolysaccharide-activated B cells result in the apoptosis of diabetogenic T cells by secretion of TGF-*β* in vitro; furthermore, administration of activated B cells to prediabetic NOD mice decreases the incidence of diabetes by inhibiting Th1 responses while not enhancing Th2 immunity to autoantigen [11]. These activated B cells generate mononuclear cell apoptosis in the spleen and provisionally dampen the activity of APCs [11]. Adoptive transfer of BCR-stimulated B cells protects NOD mice from autoimmune diabetes [12]. This protective effect requires IL-10 secretion by B cells and accompanies with decreased IFN-*γ* level and increased generation of IL-4 and IL-10 by CD4^+^ T cells [12]. Of interest, increased CD40^+^ and IL-10-competent B cells have been reported in the islets of long-term euglycemic mice. IL-10^+^ B cells show regulatory capacity to suppress T cell response to self- or islet-specific peptides in vitro and reduce antigen presentation [13]. Moreover, healthy controls exhibit a higher proportion of CD40^+^ and IL-10^+^ B cells than T1D patients [13]. These IL-10^+^, but not IL-10^−^, B cells prominently abolish T cell-mediated responses to self- or islet-reactive peptides in vitro. In line with the ex vivo results, IL-10^+^ B other than IL-10^−^ B cells alleviate T cell-mediated autoimmune disruption of islets and maintain euglycemia in NOD.SCID mice much longer [13]. Recently, the genetic/pharmacological modulation of the AID/RAD51 axis prevents NOD mice from T1D through inducing an expansion of CD73^+^ B lymphocytes, which shows regulatory activity by restraining diabetogenic T cell-mediated responses [54]. Boldison et al. [55] showed that natural protection from T1D in NOD mice is related with increased amounts of IL-10-competent B cells, while progress of T1D in NOD mice is associated with decreased numbers of IL-10-producing B cells. If activated by toll-like receptor 4, B lymphocytes from hyperglycemic mice obtain IL-10 function again and can inhibit insulin-responsive CD8^+^ T cells in a dendritic cell-dependent cell contact with Bregs and IL-10-dependent manner [55]. Genetic research of the B cell subsets in patients with T1D identifies correlation of the IL2-IL21 T1D locus with IL-10 secretion by memory B cells as well as islet-reactive CD4^+^ T cells [56].

Bregs widely defined as B10 cells in mice are CD5^+^CD1d^hi^ and produce IL-10. Recent evidence demonstrated that pDCs enhance the frequency and numbers of this population in vitro and in vivo and then reverse T1D progression in NOD mice [57]. We also found decreased B10 cells (IL-10-producing CD19^+^CD5^+^CD1d^hi^) in circulating blood of patients with T1D compared with patients with latent autoimmune diabetes in adults and patients with type 2 diabetes. Furthermore, we reported that the frequency of B10 cells is positively associated with fasting C-peptide and negatively related with hemoglobin A1c [50]. Several phenotypes of Bregs have been reported in human autoimmune disease, and these patients exhibit reduced numbers of Bregs and/or functionally impaired [58–61]. The function and phenotype of this altered Breg compartment in T1D are controversial. More recently, Wang et al. [62] reported that B10 cells in human peripheral blood belong to a CD24^hi^CD38^hi^ B cell subset. CD24^hi^CD38^hi^ B cells from healthy subjects are endowed with regulatory capacity, by inhibiting Th1 and Th17 response and converting naive T cells to Th2 cells and Tregs through an IL-10-dependent mechanism. By contrast, CD24^hi^CD38^hi^ B cells from T1D patients are decreased, produce less IL-10, and have impaired regulatory ability compared with those extracted from healthy controls. Thompson et al. [56] described the prominent reduction with age of transitional CD19^+^CD24^hi^CD38^hi^ B cells. Taken together, these data offer new insights into T-B cell interplay and suggest that Bregs may provide a novel therapeutic strategy for human T1D.

## 5. Conclusions

T1D is an organ-specific autoimmune disease resulting in the destruction of pancreatic *β* cells. B cells have been demonstrated to play a dual role in this process not only of pathogenic effect but also of immunoregulatory function in T1D. Although a transient preservation of *β* cell function is observed in treated subjects with rituximab, it does not seem to fundamentally alter the underlying pathogenesis of the disease [41]. Therefore, it requires further improvement of therapeutic efficacy. With respect to the heterogeneity of islet-infiltrating B cells [3, 4], appropriate patient phenotyping should be considered for future studies targeted to islet-infiltrating immune cells in T1D, especially taking into account age at diagnosis. Analyses of B cell communication with different T cell subsets have emphasized the multiplex roles of B cells in the humoral and cellular immune responses of T1D, via autoantibody production, antigen presentation, and cytokine secretion. Understanding these cellular interactions between B and T cells will provide vital insight into the immune pathogenesis and improve the efficacy of preventive and therapeutic strategies of human T1D. Importantly, B cell depletion combined with oral anti-CD3 prevents and reverses autoimmune diabetes in NOD mice through synergistically promoting the immunoregulation function of Tregs [63]. It will be more interesting to target autoantigen-specific rather than pan-B cell depletion while preserve Bregs to restore immune tolerance. Future studies are warranted to ascertain more specific markers and understand antigen specificity and clonality of Bregs. Transfusion of in vitro expanded autologous Bregs might offer a novel tool to therapeutic strategies for treating T1D.

## Figures and Tables

**Table 1:** The double effect of B cells on effective and regulatory T cells in nonobese diabetic mice.

Treatment	Therapeutic effect	Autoantibody production	Effective T cells	Regulatory T cells	Reference
Anti-CD20	Delay; reduce 35% diabetes onset; reverse 36% hyperglycemia in mice	Reduced	Reduced IFN-r and IL-17 production by diabetogenic CD4^+^ or CD8^+^ T cells	Expansion of CD4^+^CD25^+^Foxp3^+^ and CD4^+^CTLA4^+^ T cells	[15]

Anti-CD20	35% and 55% protection	N.D	No effect on T cell activation; impaired T cell proliferation	No effect	[16]

Anti-CD22	40% protection; 60% reversal of diabetes	N.D	Decreased proinflammatory cytokine levels and IFN-r-producing T cells	Increased percentage of CD4^+^CD25^+^Foxp3^+^ cells	[17]

BCMA-Fc	100% protection	N.D	Blunted T cell activation; decreased frequencies of pathogenic CD4^+^ and CD8^+^ CD40^+^ T cells; reduced Th1 cytokine secretion	Increased CD4^+^CD25^+^ regulatory T cells but similar frequency of CD4^+^CD25^+^ Foxp3^+^ cells	[19]

Anti-BAFF	Delay; reduce 50% and 100% diabetes incidence	Reduced	Disrupted CD4^+^ T cell activation	No effect	[18]

Lipopolysaccharide	>80% protection	N.D	Promote the apoptosis of diabetogenic T cells; suppress pathogenic Th1 immunity; no effect on Th2 responses	N.D	[11]

Anti-IgM	Delay and reduce 30-40% diabetes incidence	N.D	Suppress anti-CD3-induced splenocyte proliferation; decreased IFN-*γ* production by CD4+ T cells but increased production of IL-4 and IL-10 by CD4^+^ T cells	N.D	[12]

BAFF: B cell–activating factor; BCMA: B cell maturation antigen; IFN: interferon; IL: interleukin; N. D: not done.

## Data Availability

Data sharing is not applicable to this article as no datasets were generated or analyzed during the current study.
